# 16.4 EFFECT OF GENOTYPE AND EARLY ADVERSITY ENVIRONMENT ON DNA METHYLATION

**DOI:** 10.1093/schbul/sby014.063

**Published:** 2018-04-01

**Authors:** Darina Czamara, Polina Girchenko, Anna Suarez Figueiredo, Jari Lahti, Katri Räikkönen, Elisabeth Binder

**Affiliations:** 1 Max Planck Institute of Psychiatry; 2 Institute of Behavioral Sciences, University of Helsinki

## Abstract

**Background:**

Fetal or prenatal programming, i.e. the process in which environmental events during pregnancy are shaping and determining the development of the embryo, can be embedded by epigenetic changes including DNA methylation. Apart from environment, also the genome plays an important role and a variety of studies which identified meQTLs (methylation quantitative trait loci, i.e. SNPs which are associated with methylation levels) have been published.

**Methods:**

Focusing on variably methylated regions (VMRs), we investigated if genotype (G), prenatal environment (E) or the combination of both (GxE, G+E) best explain cordblood DNA methylation in sample of 817 Finnish neonates. Furthermore, we used an epigenetic clock predictor to evaluate if accelerated or decelerated epigenetic age was associated with prenatal environment or with childhood psychiatric problems at age 3.

**Results:**

We found that SNP genotype alone best explained methylation status in 44%, SNP x environment in 32% and SNP and prenatal environment in 24% of all VMRs. While functionally relevant meQTLs were located in close proximity to the specific CpG-site, functionally relevant SNPs involved in interaction models showed much broader peaks.

Concerning the epigenetic clock, lower gestational age was associated with maternal depression diagnosis and greater depressive symptoms throughout pregnancy. Epigenetic age deceleration was associated with pre-eclampsia. Furthermore, lower epigenetic gestational age was significantly associated with greater total and internalizing problems in boys.

**Discussion:**

Not only environment but also genotype should be considered in epigenetic studies. Furthermore, our results suggest that long-distance effects are present in GxE interactions. The epigenetic clock which mirrors prenatal environment is partially predictive for future development of the child. Lower epigenetic gestational age seems to be developmentally disadvantageous for boys, who in early childhood show greater psychiatric problems.

